# NOD1-Mediated Mucosal Host Defense against *Helicobacter pylori*

**DOI:** 10.4061/2010/476482

**Published:** 2010-07-15

**Authors:** Tomohiro Watanabe, Naoki Asano, Atsushi Kitani, Ivan J. Fuss, Tsutomu Chiba, Warren Strober

**Affiliations:** ^1^Mucosal Immunity Section, Laboratory of Host Defenses, National Institute of Allergy and Infectious Diseases, National Institutes of Health, Building 10-CRC Room 5W3940, 10 Center Drive, Bethesda, MD 20892, USA; ^2^Department of Gastroenterology and Hepatology, Kyoto University Graduate School of Medicine, 54 Shogoin Kawahara-cho, Sakyo-ku, Kyoto 606-8507, Japan

## Abstract

Infection of the stomach with *Helicobacter pylori* is an important risk factor for gastritis, peptic ulcer, and gastric carcinoma. Although it has been well established that persistent colonization by *H. pylori* is associated with adaptive Th1 responses, the innate immune responses leading to these Th1 responses are poorly defined. Recent studies have shown that the activation of nucleotide-binding oligomerization domain 1 (NOD1) in gastric epithelial cells plays an important role in innate immune responses against *H. pylori*. The detection of *H. pylori*-derived ligands by cytosolic NOD1 induces several host defense factors, including antimicrobial peptides, cytokines, and chemokines. In this paper, we review the molecular mechanisms by which NOD1 contributes to mucosal host defense against *H. pylori* infection of the stomach.

## 1. Introduction

*Helicobacter pylori *is a microaerophilic, gram-negative bacterium that colonizes human gastric mucosa [1, 2]. Although most individuals with a chronic gastric infection of *H. pylori* are asymptomatic, this bacterium causes peptic ulcer or gastric cancer in a subpopulation of susceptible hosts [1, 2]. The development of gastric disease associated with *H. pylori *infection is determined by the interplay between bacterial virulence factors and host immune responses. The *cag-*pathogenicity island (PAI) is one of the most important virulence factors of *H. pylori.* Infection with strains of *H. pylori* carrying *cag*-PAI is associated with severe gastric disease, including gastric cancer [3].

Chronic infection with *H. pylori* is characterized by a strong T helper type 1 (Th1) response in the gastric mucosa [4, 5]. Th1 cells produce IFN-*γ*-mediated mucosal host defense against this organism as shown by the fact that IFN-*γ*-deficient mice fail to eradicate the bacteria from their stomachs upon oral administration of this organism [6]. Although it is well established that the gastric mucosa of patients with *H. pylori* infection is characterized by adaptive Th1 responses, the innate immune responses leading to such Th1 responses are poorly understood. Pattern recognition molecules particularly Toll-like receptors (TLRs) play a crucial role in host defense against mucosal pathogens. TLRs are evolutionarily conserved receptors, which recognize microbial antigens and contribute to host defense by producing proinflammatory cytokines and antimicrobial peptides [7]. The activation of TLRs in antigen-presenting cells (APCs) by TLR ligands associated with *H. pylori* has in fact been shown to be involved in the generation of protective Th1 responses against *H. pylori* [8]. In addition, the neutrophil-activating protein of *H. pylori* induces Th1 responses via TLR2-mediated IL-12 secretion in APCs [9]. Thus, it is clear that the detection of *H. pylori*-associated antigens by APCs expressing TLRs plays a role in the induction of protective anti-*H. pylori* Th1 responses.

Given the fact that most gastric APCs are localized in the submucosal areas and that *H. pylori* adheres to the luminal surface of the epithelium [10], it is likely that innate immune responses by gastric epithelial cells (ECs) directly activated by the organism are involved in the immunopathogenesis of *H. pylori*-associated gastric diseases. TLR signaling may be involved in the development of *H. pylori*-associated gastric disease since patients with gastric adenocarcinoma are more likely to carry the TLR4 polymorphisms [11]. However, on the other hand, TLR signaling may not necessarily play a major role since gastric ECs have been shown to be hypo-responsive to TLR ligands [12]. Another possibility is that epithelial cell signaling occurs through Nucleotide-binding oligomerization domain, Leucine-rich Repeat-containing (NLR) proteins, that is, a rapidly emerging family of innate immune regulatory molecules that function much like TLRs but with different signaling mechanisms [13]. Nucleotide-binding oligomerization domain 1 (NOD1) is of particular relevance in this context since NOD1, a member of the NLR family proteins, recognizes small peptides derived from peptidoglycan (PGN), a component of bacterial cell walls, and is expressed in APCs and gastric ECs [14]. In addition, Viala et al. has shown that eradication of *H. pylori* requires the sensing of PGN by cytosolic NOD1 expressed in gastric ECs [15]. Since this latter discovery, several mechanisms regarding NOD1-mediated mucosal host defense against *H. pylori* have been proposed. In this paper, we focus on these mechanisms with the aim of explaining just how NOD1 signaling contributes to gastric inflammation and host defense against *H. pylori*.

## 2. Expression of NOD1

NOD1 consists of a C-terminal LRR (Leucine-rich region), a central NOD, and an N-terminal CARD (caspase-activating domain) domain [14]. Whereas TLRs are associated with the plasma membrane or endosomal vesicles, NOD1 is expressed in the cytosol [14]. NOD1 is mainly expressed by cells—APCs and ECs—that are exposed to microorganisms [14]. Importantly, most gastrointestinal cell lines and primary ECs express NOD1 [15, 16]. 

NOD1 expression is regulated by proinflammatory cytokines. IFN-*γ* activates the promoter of NOD1 via nuclear translocation of IFN-regulatory factor 1 (IRF1) to up-regulate the expression of NOD1 in intestinal ECs whereas NF-*κ*B activation by TNF does not alter the NOD1 expression in these cells [16, 17].

## 3. Signaling Pathways of NOD1

It is now established that NOD1 senses a small molecule derived from bacterial cell wall PGN. A minimum motif of NOD1 ligand is *γ*-D-glutamyl-meso-diaminopimelic acid—called iE-DAP. PGN derived from most gram-positive bacteria lacks iE-DAP. In contrast, PGN derived from most gram-negative bacteria contains iE-DAP. Thus, NOD1 functions as a sensor for gram-negative bacteria. In support of this idea, it has been shown that NOD1 participates in host defense against mucosal infection with gram-negative bacteria such as *Shigella, Escherichia coli, and H. pylori* [18, 19] although no functional mutations have been found to be associated with upper gastrointestinal diseases caused by chronic infection with *H. pylori * [20]. 

One pathway of NOD1 signaling relates to its ability to activate NF-*κ*B and MAP kinases [14]. Such signaling is initiated by the detection of NOD1 ligands by the LRR domain of NOD1 which is then followed by the recruitment of a downstream effector molecule, RICK [14]. RICK is a CARD-containing serine/threonine kinase that physically binds to NOD1 through a CARD-CARD interaction [14]. RICK then undergoes K63-linked ubiquitination and acquires the ability to recruit and activate TGF-*β*-activated kinase 1 (TAK1) [21]; the latter, in turn, initiates activation of NF-*κ*B subunits through phosphorylation and K48-linked ubiquitination of I*κ*B*α*. This sequence of events suggests that the binding of RICK to NOD1 and its K63-linked polyubiquitination is a key step in the NOD1-mediated signaling cascade with respect to responses involving NF-*κ*B and MAP kinases and, as we shall see to other responses as well. This supposition is fully supported by the fact that NOD1 responses are severely curtailed in RICK-deficient cells [14]. 

**Figure 1:**
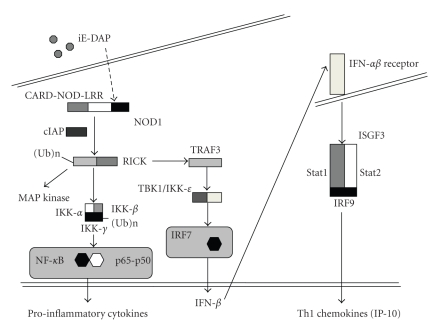
*Signaling pathways of NOD1.* NOD1 activation induces an interaction between RICK and TRAF3 that results in the production of IFN-*β* through activation of TBK1, IKK*ε*, and IRF7. IFN-*β* production leads to production of IP-10 through transactivation of ISGF3 (Stat1-Stat2-IRF9 complex). NF-*κ*B activation induced by NOD1 ligand leads to production of proinflammatory cytokines and chemokines such as IL-8.

The above NOD1 signaling pathway emphasizes the importance of ubiquitination in the signaling cascade. On the one hand, conjugation of K48-linked polyubiquitin chains with the inhibitory protein I*κ*B*α* leads to proteasomal degradation of I*κ*B*α* and the nuclear translocation of NF-*κ*B subunits such as p65 and p50. On the other hand, conjugation of K63-linked polyubiquitin chains to RICK, rather than causing RICK degradation leads to the creation of a scaffold that enables recruitment of signaling components, such as TAK1. The ligases that conjugate K63-linked polyubiquitin chains to RICK have recently been identified as the cellular inhibitors of apoptosis proteins (cIAP), cIAP1, and cIAP2 [22]. These proteins bind to NOD1 following the latters' activation by its ligands and have C-terminal RING finger domains with E3 ligase activity which then K63-ubiquitinate the RICK (Figure 1). 

As shown in recent studies [19, 23, 24], the stimulation of ECs with NOD1 ligands leads to robust production of proinflammatory chemokines. We confirmed and extended these findings with studies showing that such stimulation induced Th1 chemokines (IFN-*γ*-induced protein of 10 kDa, IP-10) in gastrointestinal ECs including freshly isolated primary ECs [17] both in the presence and absence of IFN-*γ*. Initially, we and others attributed such induction to a signaling pathway involving NF-*κ*B, along the lines described above. We noted, however, that the possible involvement of NF-*κ*B was based largely on signaling studies utilizing transfected cells, in which NOD1 and/or an NF-*κ*B reporter gene were overexpressed, rather than on studies employing cells expressing endogenous NOD1 stimulated under physiologic conditions. This introduced the possibility that signaling pathways not involving NF-*κ*B activation play an important role in NOD1 induction of chemokines in EC. In an extensive series of studies to investigate this possibility we found that indeed, stimulation of EC by NOD1 ligand and production of IP-10 was not accompanied by substantial NF-*κ*B activation, but rather by the induction of type I IFN which leads to IP-10 production by inducing the IP-10 transcription complex, IFN-stimulated gene factor 3 (ISGF3) [17]. This comes about via a unique NOD1 signaling pathway that involves initial interaction of activated RICK with TRAF3, followed by the activation of TANK-binding kinase 1(TBK1) and IKK*ε* and downstream IRF7 to induce the production of IFN-*β* and, as mentioned ISGF3 (Figure 1). ISGF3, which is a heterotrimer composed of Stat1, Stat2, and IRF9, not only acts as a transcription factor for IP-10, but also for IRF7 which induces further IFN-*β* production and further rounds of IP-10 production. Overall, these studies showed that, at least with respect to ECs, NOD1 utilizes a signaling pathway more commonly identified with cell signaling by viruses. Whether this signaling pathway also defines NOD1 function in other kinds of cells, such as macrophages, remains to be seen.

## 4. NOD1 Activation in *Helicobacter pylori* Infection

The majority of patients with *H. pylori-*associated gastritis have a higher NOD1 expression in gastric epithelial cells as compared with controls or *H. pylori*-nonassociated gastritis [20], which suggests the involvement of NOD1 signaling in the development of human gastric inflammation. In addition, animal studies demonstrate that a marked increase of bacterial load in the stomachs of NOD1-deficient mice is observed upon acute infection with *cag-*PAI-positive, but not *cag-*PAI-negative *H. pylori* as compared with NOD1-intact mice [15, 17]. These data are related to the fact that the detection of *H. pylori-*derived PGN by gastric ECs is at least partially dependent on a functional type IV secretion apparatus. Thus, as shown by Viala et al., *H. pylori *expressing functional *cag*-PAI efficiently delivered radio-labeled PGN into the ECs, whereas *H. pylori* strain 251—harboring nonfunctional *cag*-PAI—failed to deliver radio-labeled PGN [15]. In addition, these finding were supported by studies showing that infection with* H. pylori* induced IL-8 production by the gastric epithelial cell line, AGS cells, in an NOD1/*cag*-PAI-dependent manner [25]. It should be noted, however, that Kaparakis et al. have recently provided evidence for the existence of a *cag-*PAI-independent mechanism for NOD1 activation [26]. These authors purified outer membrane vesicles (OMVs) from *cag-*PAI-positive and -negative bacteria. *H. pylori*-derived OMVs containing numerous components of bacterial cell walls including PGN, induced IL-8 production by AGS cells via an NOD1-dependent and *cag-*PAI-independent fashion. OMVs activate the cytosolic NOD1 of ECs through lipid rafts. In addition, NOD1-deficient mice exhibit defective innate and adaptive immune responses to OMVs upon oral challenge with OMVs [26]. Patients infected with strains lacking a functional type IV secretion system still have inflammation and Th1 responses. These data regarding NOD1 activation by OMVs may partially explain the mechanisms by which NOD1-mediated Th1 responses against *H. pylori* are induced in the absence of functional *cag-*PAI. On the basis of this new data, further studies to determine the conditions under which NOD1 activation upon *H. pylori* infection requires functional type IV secretion apparatus are warranted.

## 5. Molecular Mechanisms of NOD1-Mediated Mucosal Host Defense against *H. pylori* Infection

**Figure 2:**
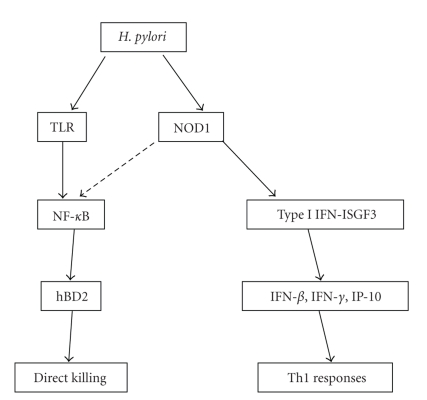
*Molecular mechanisms of NOD1-mediated mucosal host defense against H. pylori.* Infection of gastric epithelial cells with *H. pylori* activates type I IFN signaling, which leads to the generation of protective Th1 responses. *H. pylori* infection induces hBD2 production by epithelial cells mainly via TLR signaling pathways.

The generation of Th1 responses is required for mucosal host defense against *H. pylori* infection [4, 6]. Although the mechanism by which recognition of *H. pylori*-derived PGN by cytosolic NOD1 activates the protective response is not completely understood, two main models have been proposed (Figure 2). 

The first model for the role of NOD1 activation in *H. pylori* infection is based on the antimicrobial activity of defensins produced by ECs. Grubman et al. showed that *H. pylori* infection induces the production of human *β* defensin 2 (hBD2) by AGS cells in an NOD1/*cag*-PAI-dependent fashion [25]. Transfection of siRNA specific to hBD2 impairs the killing of *H. pylori*, as shown by the increased number of bacteria in culture supernatants from AGS cells infected with *cag*-PAI-positive *H. pylori*. These authors imply that NOD1 induction of hBD2 upon infection with *cag*-PAI-positive *H. pylori* may be mediated by NF-*κ*B activation, but this is unclear since NF-*κ*B activation is assessed by reporter gene assays that may not reflect endogenous NF-*κ*B regulation [25]. Consistent with these results, the expression of murine *β*-defensin 4 (an orthologue of hBD2) is markedly reduced in the gastric mucosa of NOD1-deficient mice as compared with NOD1-intact mice [27]. These data regarding hBD2 induction by NOD1 suggest that NOD1 mediates host defense by the direct killing of *H. pylori* through induction of hBD2. However, the contribution of NOD1-induced defensins to the generation of Th1 responses remains unknown.

The second model for the role of NOD1 activation in *H. pylori* infection is based on the production of type I IFN and activation of the ISGF3 signaling mediated by NOD1. As we described in the previous section regarding NOD1 signaling pathways, the stimulation of gastrointestinal ECs including primary cells with NOD1 ligands leads to a robust production of IFN-*β* and the subsequent induction of ISGF3 [17]. We therefore addressed the role of this new signaling pathway in *cag*-PAI-positive *H. pylori *infection. In *in vitro* studies we found that *H. pylori* infection of AGS cells led to a massive increase of IFN-*β* and IP-10 production, which was accompanied by the activation of both Stat1 and Stat2, suggesting that cag^+^ *H. pylori* organisms do indeed activate epithelial cells via the IFN-*β*-ISGF3 pathway. Although *H. pylori* infection of the AGS cells results in NF-*κ*B activation, NF-*κ*B activation induced by *H. pylori* infection is independent of NOD1. The infection of AGS cells in the presence of siRNA specific for NOD1 resulted in the impaired nuclear translocation of Stat1 and production of IFN-*β* and IP-10, but unchanged nuclear translocation of NF-*κ*B subunit, p65 [17]. These results are consistent with previous studies of Hirata et al. [28], who showed that *H. pylori* organisms can activate NF-*κ*B in epithelial cell lines through MyD88-dependent mechanisms, but not NOD1-dependent mechanisms, and Viala et al. who reported that primary gastric epithelial cells infected with *H. pylori* produce chemokines in the absence of the nuclear translocation of NF-*κ*B p65 [15]. 

In further in vivo studies of NOD1 activation during* H. pylori* infection we found a marked increase of bacterial load in the stomachs of NOD1-deficient mice as compared with NOD1-intact mice. Furthermore, this was associated with reduced production of type I IFN-ISGF3-associated cytokines and chemokines such as IFN-*β*, IP-10, and IFN-*γ* rather than NF-*κ*B-associated cytokines such as TNF. Thus, the activation of type I IFN and ISGF3 signaling via NOD1 is responsible for the generation of protective Th1 responses against *H. pylori *[17]. This idea was confirmed by the fact that gene silencing of Stat1, a component of ISGF3, increases the bacterial burden in the stomach of NOD1-intact mice due to impaired Th1 and type I IFN responses. Therefore, it is likely that NOD1-mediated type I IFN production and ISGF3 activation provide protective Th1 responses upon infection with *cag*-PAI-positive *H. pylori*. In support of this model, IP-10 expression is observed in the gastric mucosa of patients with chronic *H. pylori *infection [5]. 

Recent studies addressing the role of NOD1 in mucosal host defense against *H. pylori *have been focusing on acute innate responses as assessed by cell-culture or animal infection models. Thus, it is unclear how NOD1 activation is involved in the development of various gastric diseases associated with chronic *H. pylori* infection. In this regard, IL-17 may play an important role in the chronic inflammatory responses to *H. pylori *infection as shown by the overproduction of this cytokine in *H. pylori*-infected human gastric mucosa [29]. In addition, vaccination of mice against *H. pylori *results in efficient eradication due to enhanced IL-17 production in the gastric mucosa [30, 31]. It is possible that NOD1 activation in response to *H. pylori* infection is involved in the generation of gastric Th17 responses since NOD1 triggering is required to instruct the onset of both Th1 and Th17 responses [32].

## 6. Conclusions

Infection with *cag*-PAI-positive *H. pylori* leads to the activation of NOD1 in gastric ECs and to the induction of several host defense factors including hBD2, IFN-*β*, and IP-10, all of which regulate bacterial growth. Recent studies have shown that NOD1 activation is mediated in ECs largely, if not entirely, by NOD1 induction of IFN-*β* and thus a signaling pathway ordinarily activated by viral infection. This has important implications for our views on how the mucosal system utilizes innate immune mechanisms to deal with chronic bacterial infections. Interestingly, proinflammatory cytokine responses induced by TLR ligands are markedly enhanced in the presence of NOD1 ligands. Therefore, it is possible that the synergistic activation of NOD1 and TLRs in gastric APCs also plays a role in mucosal host defense against this organism. Thus, future studies addressing whether or not NOD1 expression in APCs contributes to host defense against *H. pylori* will be of great interest.

## Abbreviations

APCs: Antigen-presenting cells
CARD: Caspase-activating domain
cIAPs: cellular inhibitor of apoptosis proteins
ECs: Epithelial cells
hBD2: human *β* defensin 2
IP-10: IFN-*γ*-induced protein of 10 kDa
IRF: IFN-regulatory factor
ISGF3: IFN-stimulated gene factor 3
LRR: Leucine-rich repeats
NLR: Nod-like receptor
NOD1: Nucleotide-binding oligomerization domain-1
OMVs: Outer membrane vesicles
PAI: Pathogenicity island
PGN: Peptidoglycan
TAK1: TGF-*β*-activated kinase 1
Th1: T helper type 1
TLRs: Toll-like receptors.
